# The type VI secretion system of *Xanthomonas phaseoli* pv. *manihotis* is involved in virulence and in vitro motility

**DOI:** 10.1186/s12866-020-02066-1

**Published:** 2021-01-06

**Authors:** Nathaly Andrea Montenegro Benavides, Alejandro Alvarez B., Mario L. Arrieta-Ortiz, Luis Miguel Rodriguez-R, David Botero, Javier Felipe Tabima, Luisa Castiblanco, Cesar Trujillo, Silvia Restrepo, Adriana Bernal

**Affiliations:** 1 Department of Biological Sciences, Universidad de los Andes, Bogotá, Colombia; 2 Institute for Systems Biology, Seattle, WA USA; 3 Department of Microbiology and Digital Science Center (DiSC), University of Innsbruck, Innsbruck, Tyrol Austria; 4 Botany and Plant Pathology Department, Oregon State University, Corvallis, OR USA

**Keywords:** Type VI secretion, Hcp, Vgr, IcmF, ClpV, *Xanthomonas*, bacterial pathogenesis

## Abstract

**Background:**

The type VI protein secretion system (T6SS) is important in diverse cellular processes in Gram-negative bacteria, including interactions with other bacteria and with eukaryotic hosts. In this study we analyze the evolution of the T6SS in the genus *Xanthomonas* and evaluate its importance of the T6SS for virulence and in vitro motility in *Xanthomonas phaseoli* pv. *manihotis* (*Xpm*), the causal agent of bacterial blight in cassava (*Manihot esculenta*). We delineate the organization of the T6SS gene clusters in *Xanthomonas* and then characterize proteins of this secretion system in *Xpm* strain CIO151.

**Results:**

We describe the presence of three different clusters in the genus *Xanthomonas* that vary in their organization and degree of synteny between species. Using a gene knockout strategy, we also found that *vgrG* and *hcp* are required for maximal aggressiveness of *Xpm* on cassava plants while *clpV* is important for both motility and maximal aggressiveness.

**Conclusion:**

We characterized the T6SS in 15 different strains in *Xanthomonas* and our phylogenetic analyses suggest that the T6SS might have been acquired by a very ancient event of horizontal gene transfer and maintained through evolution, hinting at their importance for the adaptation of *Xanthomonas* to their hosts. Finally, we demonstrated that the T6SS of *Xpm* is functional, and significantly contributes to virulence and motility. This is the first experimental study that demonstrates the role of the T6SS in the *Xpm*-cassava interaction and the T6SS organization in the genus *Xanthomonas*.

**Supplementary Information:**

The online version contains supplementary material available at 10.1186/s12866-020-02066-1.

## Background

A large number of Gram-negative bacteria use the type VI secretion system (T6SS) to transport proteins across the bacterial cell envelope. This versatile protein secretion system seems to be involved in a variety of cellular processes in bacteria, including antibacterial activity, biofilm formation and interactions with eukaryotic hosts. Thus, the T6SS may confer a competitive advantage in multi-species environments. The T6SS is responsible for antagonism towards potentially competing bacteria by direct injection of protein effectors in species such as *Pseudomonas aeruginosa* [1, 2]^,^ *Salmonella typhimurium* [3] and *Agrobacterium tumefaciens* [4]. In *Acidovorax citrulli* [5] and *Burkholderia cenocepacia* [6] the T6SS has been implicated in biofilm formation as well*.*

In addition, the T6SS also participates in the interactions of pathogenic and commensal bacteria with their eukaryotic hosts. For example, in *P. aeruginosa*, two out of the three T6SS clusters are important in virulence against eukaryotic cells [7, 8]. This system is also involved in cell to cell signaling and communication. *Vibrio cholerae* uses the T6SS to induce changes in the host cellular behavior that reduce the population of other, potentially competing, bacteria [9]. In plant pathogens, such as *Pantoea ananatis,* the T6SS plays a key role in pathogenesis and bacterial competition [10]. Despite its importance, information about the functions of this system in plant pathogens remains scarce.

The T6SS injects diverse effector proteins into cells by contracting a spike-containing inner tube that perforates the membrane of target cells [11]. The system is typically encoded by a set of fifteen to twenty genes. However, bacterial genomes encoding for T6SSs share a group of thirteen fundamental core genes [12]. One of the most important proteins in this machinery is ClpV, an ATPase that forms a hexameric complex that provides the energy required for secreting T6SS substrates [13, 14]. Aditionally, the intracellular multiplication protein F (IcmF or TssM*)*, is essential for the secretion of the haemolysis-corregulated protein (Hcp) [15–17]. Hcp and VgrG (Valine-Glycine repeats G), are both effector proteins and important parts of the structural machinery of the T6SS in *A. tumefaciens* [18]. Hcp and VgrG show structural homology to proteins found in the tail structures of bacteriophages, suggesting an evolutionary relationship between the T6SSs and the cell-puncturing machinery of family bacteriophages belonging to the *Myoviridae* [19, 20].

The T6SS components are encoded in gene clusters that vary in organization and frequency. For example *P. aeruginosa* has three T6SS clusters [8] while *Burkholderia thailandesis* has five different clusters [21]. Boyer and collaborators [12] found that *Xanthomonas axonopodis* and *Xantohomonas campestris* both have two complementary T6SS loci. Similarly, *Xanthomonas euvesicatoria* 85–10 (*Xeu*) has two T6SS loci or clusters with 15 conserved components [22]. The T6SS has been partially characterized, through bioinformatics, in *Xeu*, *Xanthomonas vesicatoria* strain 1111 (ATCC 35937: *Xv*) and *Xanthomonas perforans* strain 91–118 (*Xp*) [23]. Mutants have been generated for genes *vgrG* and *clpV* in *Xeu* [22]. But, no change in virulence was reported for those mutant strains. Moreover, in *Xanthomonas citri*, the T6SSmediates resistance to *Dictyostelium* predation [24]. Overall, the T6SS of members of the *Xanthomonas* genus need further characterization.

*Xanthomonas phaseoli* pv. *manihotis* (*Xpm*) is the causal agent of cassava bacterial blight, an economically important disease in Africa and South America, causing losses that may reach up to 100% after three cycles of cassava production [25]. This local and systemic pathogen induces a wide combination of symptoms such as angular leaf spots, blight, wilting, dieback, gum exudation and vascular necrosis [25]. Here, we report a bioinformatic study of the organization of the T6SS cluster in the genus *Xanthomonas.* We also report on the importance of this system for bacterial virulence and in vitro motility.

## Methods

### Determining the core components of the T6SS in Xanthomonads

**Fig. 1:**
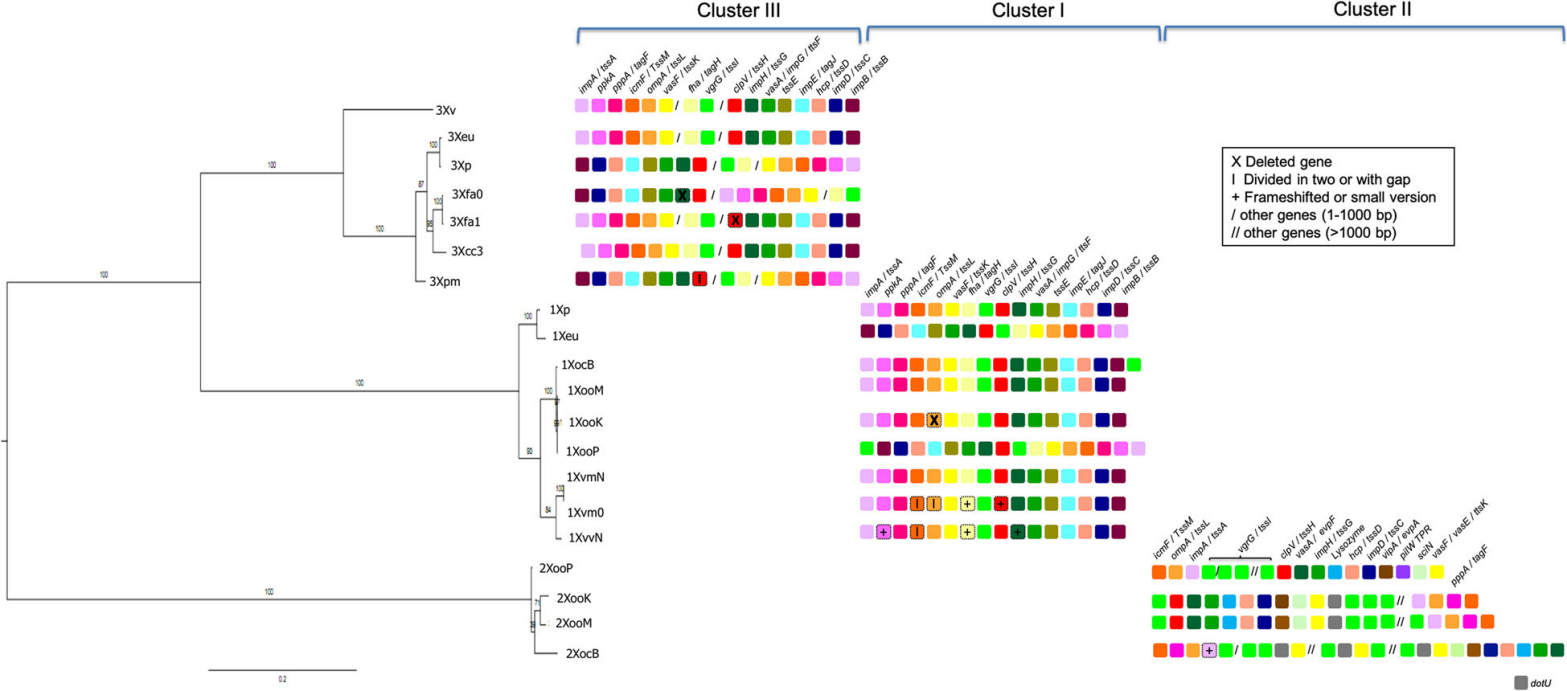
Correlation between phylogeny and T6SS organization in the genus *Xanthomonas*. Three distinct T6SS cluster were identified in the genus *Xanthomonas* (see Table S5: Structural genes of type VI secretion system of *Xanthomonas*). The phylogenetic tree was constructed using maximum likelihood. Support values for each clade (estimated with 1000 bootstraps) are shown. The tree shows two main groups, where *X*. *vesicatoria* (3Xv), *X. euvesicatoria* str. 85–10 (3Xeu), *X. perforans* 91–118 (3Xp), *X. fuscans* subsp. *aurantifolii* str. ICPB10535 (Xfa0), *X. fuscans subsp. aurantifolii* str. ICPB 11122 (Xfa1), *X. citri* subsp*. citri* str. 306 (3Xcc), *X. phaseoli* pv. *manihotis* str. CIO151 (3Xpm), *X. vasicola* pv. musacearum str. NCPPB 4380 (XvmN), *X. vasicola* pv. *musacearum* JCVI (Xvm0) and *X. vasicola* pv. *vasculorum* str. NCPPB 702 (XvvN) are grouped together. While the type II cluster of *X. oryzae* pv. oryzicola str. BLS 256 (XocB), *X. oryzae* pv. *oryzae* str. PXO99 (XooP), *X. oryzae* pv. *oryzae* str. MAFF 311018 (XooM) and *X. oryzae* pv. *oryzae* str. KACC 10331 (XooK) are clustered into a second group. Core T6SS conserved genes are depicted on the right column with small squares. The color assigned to each gene was consistently used among all T6SS clusters

A bioinformatic search for genes involved in the T6SS machinery was performed by selecting a group of genes of *P. aeruginosa* [15], and performing a TBLASTN search with the BLOSUM62 matrix [26] against the genomes of *Xanthomonas citri* subsp. *citri* strain 306 (*Xcc3*)*, Xeu* and *Xpm* (Additional file: Table S1)*.* Additionally, genes identified in *Xpm* were confirmed as orthologs using reciprocal best hit. In order to identify all components of T6SS components in other xanthomonads, we used the same genes from *P. aeruginosa* and performed a BLASTP search with the BLOSUM62 matrix [26] against the genomes of *Xanthomonas oryzae* pv. *oryzae* (*Xoo*), *Xanthomonas campestris* pv. *campestris* (*Xcac*) and *Xanthomonas albilineans* (*Xalb*) (Additional file: Table S1). In both cases, a homolog was considered as significant if the BLAST e-value was < 10^− 20^ and the amino acid identity was at least 30%. A subsequent search for orthologs with ORTHOMCL [27] confirmed the results [28, 29]. To detect signatures of Horizontal Gene Transfer, a search for genomic islands and insertion sequences was performed using Alien Hunter [30] and IS finder [31], respectively. These results allowed the reconstruction of the T6SS clusters of *Xcc3*, *Xeu* and *Xpm* (Fig. 1). The resulting T6SS gene clusters were used in BLAST searches against the other *Xanthomonas* genomes (listed in Table S1). In addition, conservation of the T6SS in *Xpm* was assessed by BLASTN searches default parameters [26] for the 65 *Xpm* strains reported by Bart and collaborators [32].

### Phylogenetic reconstruction

The orthologous gene sequences were aligned using MUSCLE 3.8 [33]. All core ortholog alignments were concatenated into a super matrix in Geneious [34] for phylogenetic reconstruction. The phylogenetic tree was constructed using a maximum likelihood approach in RAxML V.7.2.8 [35] using the GTR + G + I model of nucleotide evolution and partitions per gene in the different clusters. Support values for phylogeny branches were estimated by means of 1000 replicates of bootstrap in RAxML [35].

### Cellular localization, protein family and motif prediction for T6SS genes of *Xpm*

The cellular localization of each protein in the dataset was determined using PSORT [36], with options for Gram-negative bacteria. To determine transmembrane region and their orientation, TMpred was used [37]. The databases InterPro [38], CDD [39], and ProDOM [40, 41] were used for protein domain identification. The Pfam database [41] was used to assign protein families. The presence of known protein motifs in the T6SS components was determined using Motif Finder [42] and MOTIF Search in TRANSFAC databases [43].

### Bacterial strains and growth conditions

The strains, plasmids and primer sequences used in this study are described in Tables S2 and S3. *Escherichia coli* strain DH5α was grown on LB at 37 °C and the *Xpm* strain CIO151 was grown on LPGA (5 g yeast extract, 5 g dextrose, 5 g peptone and 15 g agar per liter of distilled water) at 28 °C. For inoculation assays, *Xpm* cells were grown for two days in LPGA agar medium with the appropriate antibiotics. Cells were subsequently grown overnight in Phi broth (5 g yeast extract, 5 g dextrose, 5 g casamino acids and 15 g agar per liter of distilled water) at 28 °C with the appropriate antibiotics. To adjust the cell suspensions to an OD_600nm_ of 0.2, overnight cultures were harvested by centrifugation at 14000 rpm for 2 min and re-suspended in 10 mM MgCl_2_ without antibiotics. In vitro growth of *Xpm* strains was measured in Phi broth with the appropriate antibiotics at 28 °C and shaking at 200 rpm for 38 h. Three independent measurements were performed.

### Swimming motility assays

Swimming motility of *Xpm* strains was evaluated by measuring the motility diameter of in LPGA medium with 0.3% agar. Swimming plates were inoculated with cultures grown overnight and adjusted to an OD_600nm_ of 0.3. They were subsequently incubated at 25 °C for 24 and 48 h. Two independent experiments were performed with four replicates each.

### Generation of knockout mutants of *vgrG*, *clpV*, *icmF* and *hcp*

Homologous recombination by single crossing-over was used to generate mutants of these genes in *Xpm*. A fragment of nearly 400 bp from the central region of each gene was amplified by PCR. A 25 μl reaction was performed including 1X Buffer, 2 mM MgCl_2_, 0.2 mM dNTPs, 0.2 mM of each primer, 10–50 ng of DNA of *Xpm* and 2 U of *Taq* polymerase (Invitrogen Corp.). The amplification proceeded with an initial denaturation step of 5 min at 95 °C, followed by 35 cycles of 45 s at 95 °C, 45 s at annealing temperature (Additional file: Table S3) and an extension time of 1 min per Kb of expected product at 72 °C. The obtained fragments were subcloned in pENTR™/ D-TOPO® (Invitrogen Corp., Grand Island, New York, USA). The clone was digested with the enzyme *Eco*RI and the resulting fragment was inserted in the suicide vector pAC3.1 [44]; and transformed into *E. coli* DH5α cells. The colonies were selected on LB plates + kanamycin (50 μg/ml) + chloramphenicol (25 μg/ml). Insertion of the fragment in the vector was confirmed by PCR (Additional file: Table S3) and sequencing (Macrogen Inc. Korea). The insertion of each mutagenesis fragment in *Xpm* was performed by triparental mating and the resulting colonies were confirmed by PCR and sequencing (Macrogen Inc., Korea). For the PCR confirmation, extension and nested primers were designed (Additional file: Table S3).

### *In planta* virulence assay

The virulence of the four generated mutants of *Xpm* was assayed on susceptible cassava plants (HMC-1 and MCOL2215). We employed two inoculation methodologies to determine differences between the pathogenicity of wild type and mutant strains: the first consisted on making 2 mm diameter perforations on the leaflets, as previously described by Restrepo and collaborators [45]. These perforations were inoculated with 10 μL of liquid bacterial suspension in 10 mM MgCl_2_ at an OD_600nm_ of 0.2 (10 [8] CFU/ml). Three independent experiments were performed with five replicates each. To measure the lesion area, we used the ImageJ package [46] and a one-way analysis of variance (ANOVA) test was performed to validate statistically significant differences between wild type and mutant bacteria. The second inoculation method was the leaf clipping method [47] that consisted of cutting one to two cm from the tip of the leaves with scissors previously dipped in the inoculum at a concentration of 0.2 OD_600nm_. Three independent measurements were performed. The onset of symptoms was monitored until day 15 post inoculation. Dilution plating was performed for each bacterial suspension in order to ensure the presence of the bacterium and to measure the concentration of the initial inoculum. *Xpm* CIO151Δ*hrpX* mutant, where secretion of effector proteins by the T3SS has been abrogated rendering the strain non-pathogenic, was used as a negative control.

## Results

### Structural genes and phylogenetic tree of the T6SS in the genus *Xanthomonas*

The T6SS of *P. aeruginosa* has been well studied both at the bioinformatic and experimental levels, and its importance in pathogenicity and in the interaction with other bacteria has been demonstrated [1, 15]. We used the T6SS genes of *Pseudomonas aeruginosa* PAO1 as references to identify the T6SS genes in 44 out of the 60 evaluated genomes of the genus *Xanthomonas*. Our BLASTP and ORTHOMCL analyses (see methods) show that the reconstructed T6SS clusters in *Xanthomonas* contain between twelve and sixteen genes. For further analyses, we only considered the genomes of species that had at the minimum set of genes required for T6SS functionality [48]. Therefore, we selected 14 representative genomes within the *Xanthomonas* genus (Additional file: Table S4 bold letter) to perform phylogenetic analyses that offer insights into the evolutionary paths and diversity in organization of the T6SS in this group of plant pathogens (Fig. 1). We identified each of the genes encoded in the T6SS in these genomes by OrthoMCL and BLASTP (Additional file: Table S5), and detected a subset of the genes in additional genomes (*Xanthomonas cassavae* str. CFBP 4642, *Xanthomonas perforans* str 91–18 and *Xanthomonas axonopodis* str.29) using EDGAR2.0 [49] (Additional file: Table S6). These analyses suggest that the T6SS is present in a widespread array of species in *Xanthomonas*, pinpointing at the biological importance of this cluster in this genus.

Among the *Xanthomonas* species with putative functional T6SS, we found three different types of clusters (referred to I, II and II in Fig. 1, additional file: Table S4, Table S5). Remarkably, the T6SS cluster are not the result of simple duplication events. For example, the *Xoo* strains have two clusters, one of which follows the phylogeny of the species, being distantly related form the *phaseoli* clade. However, the second cluster forms a monophyletic cluster with one of the copies from *X. euvesicatoria* and *X. perforans*. This implies that the clusters have a recent common origin and could have been horizontally acquired. We observed conserved synteny in the organization of Cluster III of *Xv*, *Xeu*, *Xfa1* and *Xcc3* (all members of *axonopodis* clade [28, 29]). This suggests that a common origin of the clusters precedes the divergence of the pathovars. In summary, there is evidence for both vertical and horizontal inheritance of the T6SS clusters in *Xanthomonas*.

In agreement with previous reports [23, 24], transcriptional regulators (LysR, TssB and TssA) were detected at the boundaries of some of the reconstructed T6SS clusters (I and III). The presence of noncoding RNAs in Cluster I suggests additional post-transcriptional regulation. Notably, we detected transcriptional regulators of the LysR family as a new class of regulators for Cluster I. The identified regulators may conditionally act individually or in combination to regulate their target clusters. No regulators were identified for type II clusters. Surprisingly, transcriptional regulators of the AraC family, previously propopsed as a characteristic feature of type III clusters [23], were not detected in the clusters of *Xfa* and *Xcc*.

Pseudogenes [50] were noted in the T6SS clusters of some of the genomes analyzed (Fig. 1, additional file: Table S5). We considered some proteins with an early stop codon that were divided in two parts as well as other genes that had shorter versions or frameshifts (Fig. 1). *fha*, *clpV* and *impH* were among the truncated genes in the clusters I of *Xvm0* and *XvmN*. Since Fha is a target for TagF involved in posttranslational regulation of *P. aeruginosa* and *A. tumefaciens* [51], this process may be affected in these strains and they might have an alternative type of regulation, which would need to be experimentally determined. Other divided genes of the T6SS clusters include: *icmF* in *Xvm0* and *XvmN* (cluster I), *ompA* in *Xvm0* (cluster I), and *clpV* in *Xpm* (cluster III) (Fig. 1). Experimental procedures are necessary to determine the function of these genes in the *Xanthomonas* T6SS. Sixteen *Xanthomonas* strains did not contain the core genes of the T6SS; some were *Xanthomonas campestris* pv. *campestris* (strain 8004, strain ATCC33913 and strain B100), *Xanthomonas campestris* pv. *armoraciae* str. 756C and *Xanthomonas albilineans* and were therefore considered as T6SS-depleted.

### *Xpm*, *Xeu* and *Xcc3* show similarity in the organization of the T6SS gene clusters

**Fig. 2:**
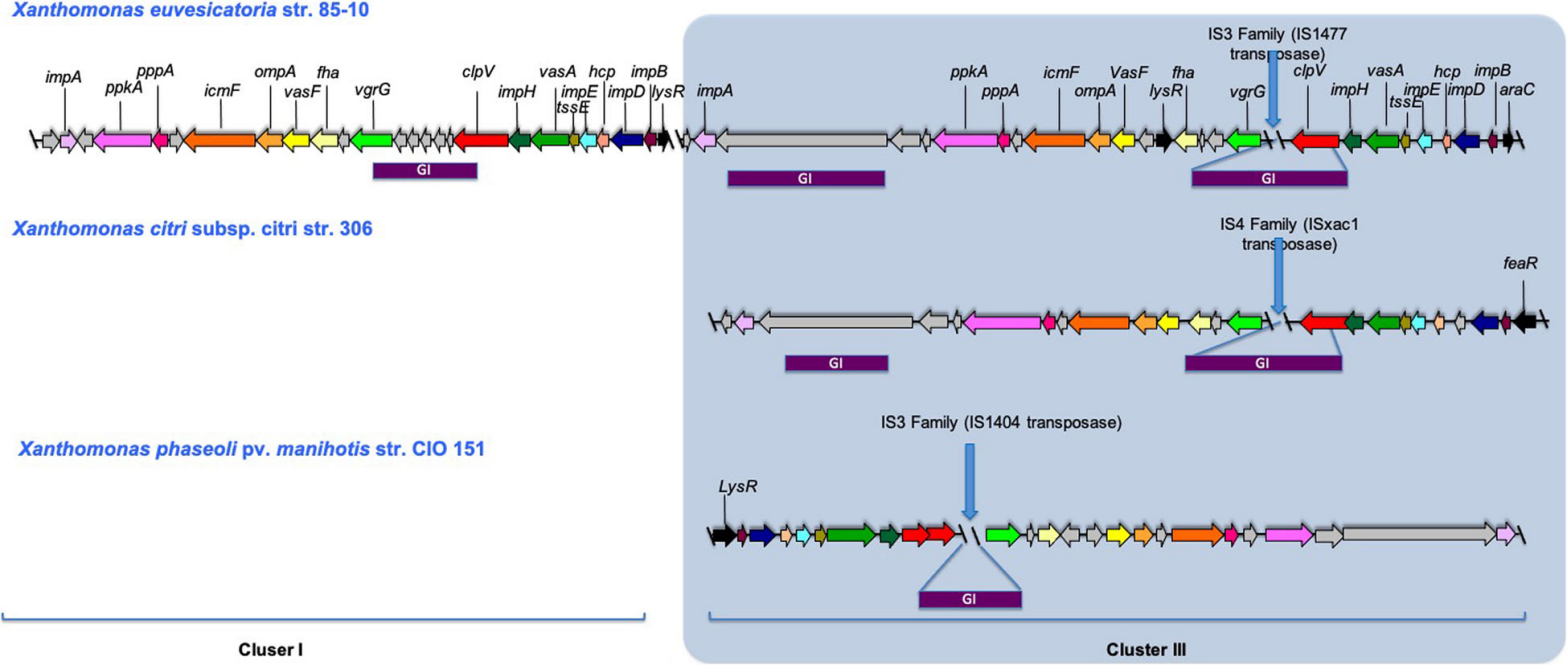
Comparative diagram of the T6SS Cluster of *Xeu*, *Xcc* and *Xpm*. T6SS cluster of *Xanthomonas euvesicatoria* str. 85–10, *Xanthomonas citri* subsp. *citri* str*.* Three hundrred six and *Xanthomonas phaseoli* pv. *manihotis* str. CIO151 are depicted. Gene names follow the nomenclature for *P. aeruginosa* and *V. cholerae* O1 T6SS genes. Arrows with the same color indicate orthologs. Blue vertical lines indicate insertion sequences (IS). Purple horizontal lines indicate genomic islands (GI), as determined by Alien Hunter [30]. Arrows with the same color show gene orthologs

A comparative analysis was performed between *Xpm* and its closest relatives with completely sequenced genomes, *Xeu* and *Xcc3* [28]. Figure 2 shows the presence of two T6SS clusters (containing the same genes in different order) of *Xeu*, In contrast, *Xpm* and *Xcc3* only have a single T6SS cluster (cluster III), orthologous to cluster III of *Xeu*.

**Fig. 3:**
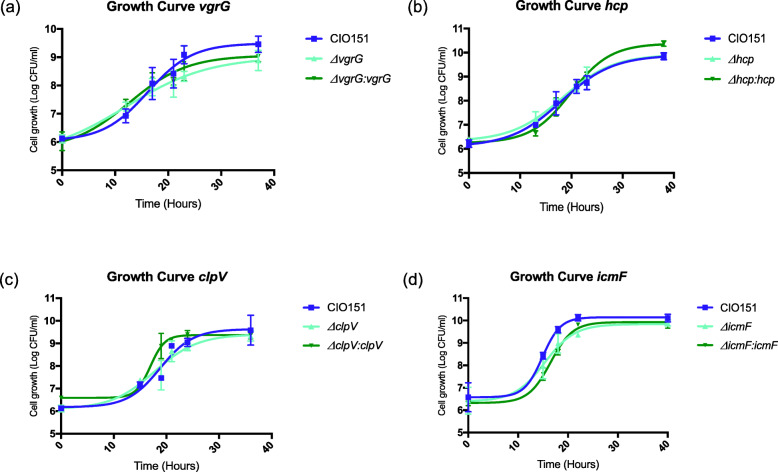
In vitro growth of *Xpm* T6SS mutants and complemented strains. Growth measurements started with a bacterial suspension of 0.2 OD_600nm_ and were evaluated from 0 h, six times, until 38 h. The wild-type CIO151 (WT) strain was used as the control (pME6010). **a** Growth curve of the WT, Δ*vgrG (*pME6010) and complemented *ΔvgrG (*pBAV226: *vgrG*) strains. **b** Growth curve of the WT, Δ*hcp (*pME6010) and complemented Δ*hcp* (pBAV226: *hcp*) strains. **c**. Growth curve of the WT, Δ*clpV (*pME6010) and complemented Δ*clpV (*pBAV226: *clpV*) strains. **d**. Growth curve of the WT, Δ*icmF (*pBAV226), and complemented Δ*icmF (*pBAV226: *icmF*) strains. Values are means from three repetitions, and vertical bars represent ± Standard Error Media

**Fig. 4:**
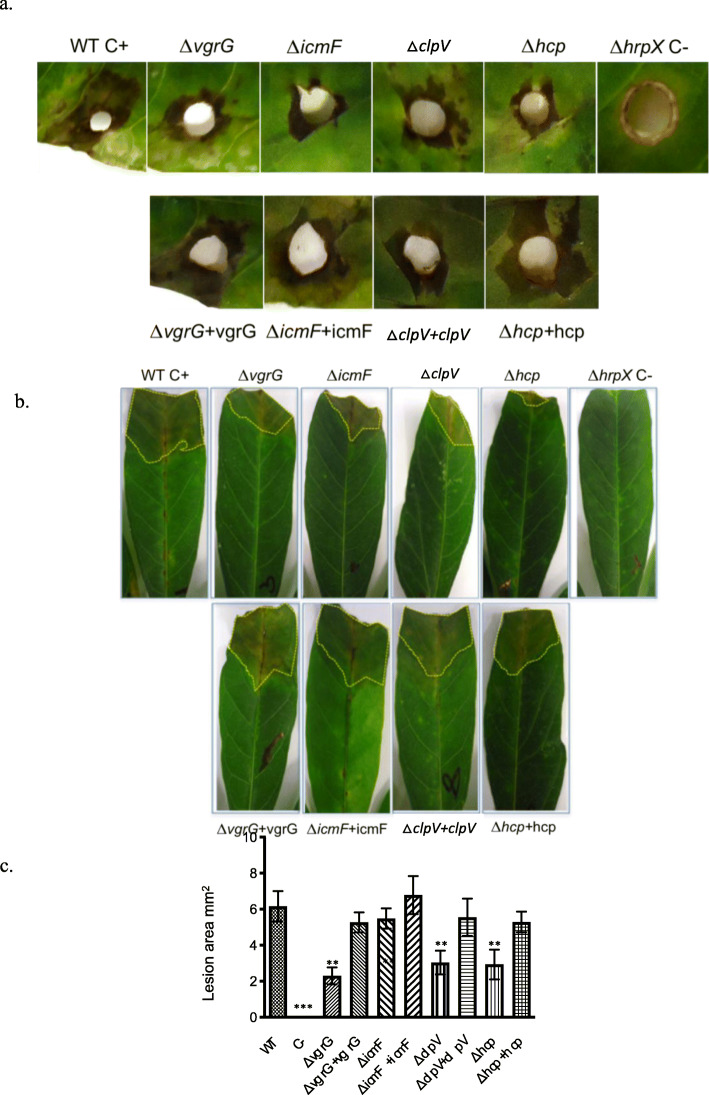
Mutations in *vgrG*, *clpV and hcp1* decrease the virulence of *Xanthomonas phaseoli* pv. *manihotis*. Images show susceptible cassava leaves inoculated with the *Xpm* strains CIO151 (positive control), Δ*vgrG*, Δ*clpV,* Δ*icmF,* Δ*hcp* and *ΔhrpX* (negative control) strains. **a**. Symptoms obtained at 15 dpi (days post inoculation) using a hole inoculation assay. Similar results were obtained in two independent biological replicates. **b**. Symptoms obtained at 15 days post inoculation (dpi) using inoculation with scissors. **c**. Average lesion area produced by the *Xpm* strains in evaluation at 15 dpi using a hole inoculation assay (from (**a**)). Values are the means ± standard deviations from five replicates. One-way analysis of variance (ANOVA) test was performed. ** Significant difference (*p*-value < 0.01)

To determine if the T6SS is conserved among members of the *Xpm* pathovar, or is exclusively present in the CIO151 strain, we searched for the T6SS genes in the genomes available for this pathovar [32]. We performed a BLASTN analyses with each T6SS gene of CIO151 against the other 64 genomes of *manihotis* pathovar (data not shown). We found the 16 T6SS genes are conserved among the analyzed genomes, with 75 to 100% identity at the nucleotide level. We found that *clpV* is divided in two parts due to a stop codon in the middle of the two AAA-ATPase domains. Nonetheless, because the stop codon does not truncate any of the protein domains (Additional file: Table S7), the two ClpV fragments may interact to create a functional protein complex (Figs. 3 and 4).

The cluster of orthologous groups (COG), together with the results obtained from our phylogenetic results discussed above, suggest Horizontal Gene Transfer (HGT) events as the probable origin of the T6SS in *Xanthomonas*. Thus we searched for genomic islands (GI), insertion sequences (IS), deviations in the GC content, and tRNA genes surrounding the T6SS clusters [12]. The positions and types of identified GI and IS were not exactly the same in clusters I and clusters III of the three organisms (*Xpm*, *Xeu* and *Xcc3*, Fig. 2 and additional file: Table S8). However, GIs ere consistently located between the *vgrG* and *clpV* genes. The IS within the T6SS clusters of *Xeu* and *Xpm* belong to the IS3 family, while the IS in the *Xcc3* cluster is part of the IS4 family [31] (Fig. 2 and additional file: Table S8). In contrast, no significant difference in GC content (with respect to the average GC content of the corresponding genomes) was detected int the T6SS cluster of *Xeu*, *Xcc3*, and *Xpm*. Hence, there are some characteristics of HGT for these clusters, but if these events occurred, there has been enough time to adapt these clusters to the rest of the genome.

### Mutations in *vgrG*, *clpV* and *hcp* decrease the virulence of *Xpm* on susceptible cassava plants

To determine whether the T6SS contributes to the virulence of *Xpm*, we constructed and then inoculated *vgrG*, *clpV, icmF* and *hcp* single deletion mutants into susceptible cassava plants. Initially, in vitro growth assays were performed for each mutant strain, to determine whether the introduced mutations decrease the general fitness of the pathogen (Fig. 3). The mutants were complemented with the wild type genes in all cases to restore the genotype. The resulting *vgrG*, *clpV* and *hcp* mutant strains did not show significant differences in fitness with respect to the wild type control (paired t-test *p*-values = 0.14, 0.48 and 0.15, respectively). *icmF* mutants showed a small decrease in growth in vitro at 18 h with respect to the wild type strain (paired t-test p-value = 0.02) but the effect was undetectable at later time points.

These results suggest that while mutations in *vgrG*, *clpV and hcp* do not affect the ability of *Xpm* to grow in vitro, mutation in *icmF* has a slight effect on the growth of this pathogen*.*

We then tested the mutants for their ability to cause disease in susceptible cassava plants. The *vgrG*, *clpV* and *hcp* mutant strains were able to produce symptoms on leaves of susceptible cassava. Therefore, we conclude that these genes are not required for full pathogenicity (Fig. 4). However, a decrease in symptoms was observed for CIO151Δ*vgrG*, CIO151Δ*clpV*, and CIO151Δ*hcp* strains at 15 days post-inoculation by two different methods of inoculation (Fig. 4a and Fig. 4b). For the hole-inoculation method, the lesion area was measured with the program ImageJ [46] and statistically significant differences when compared to the wild type were observed (ANOVA *p*-value> 0.01), as show in Fig. 4c. The phenotype was complemented when mutants were transformed back with their respective wild type gene (Fig. 4c). Together, these results suggest that the genes *vgrG*, *clpV* and *hcp* are required for maximal aggressiveness of *Xpm* on susceptible cassava plants.

### *clpV* deletion decreases *Xpm* motility

**Fig. 5:**
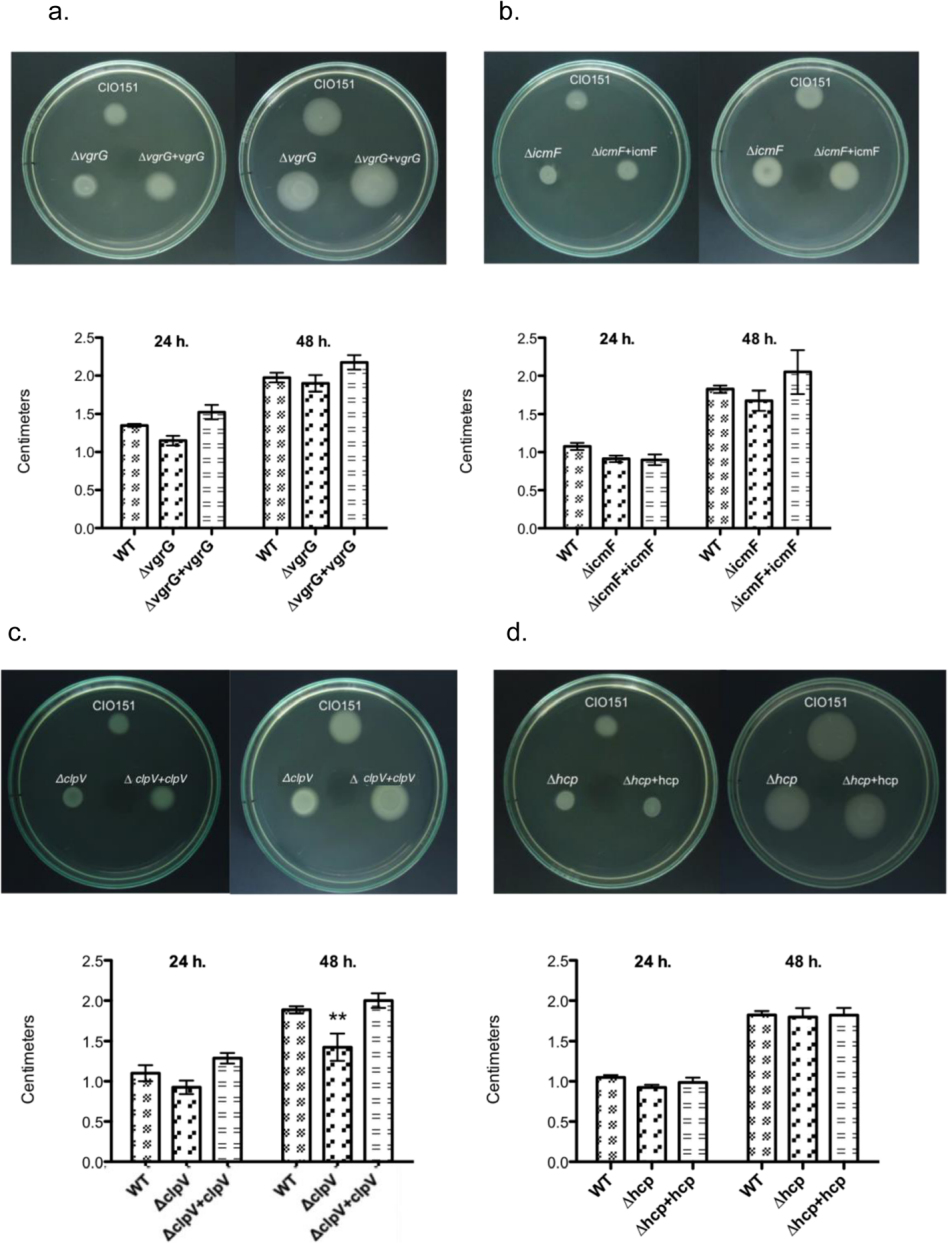
Swimming motility assays of *X. phaseoli* pv. *manihotis* CIO151 and T6SS mutants. **a**. Δ*vgrG* (pME6010) and *ΔvgrG + vgrG* (pBAV226: *vgrG*) correspond to the *vgrG* deletion and the complemented *vgrG* deletion strains, respectively*.* **b**. Δ*icmF* (pBAV226) and Δ*icmF* (pBAV226: *icmF*) correspond to the *icmF* deletion and the complemented *icmF* deletion strains*.* **c***.* Δ*clpV* (pME6010) and Δ*clpV* (pBAV226: *clpV*) correspond to the *clpV* deletion and the complemented *clpV* deletion strains*.* **d**. Δ*hcp* (pME6010) and Δ*hcp* (pBAV226: *hcp*) correspond to the *hcp* deletion and the complemented *hcp* deletion strains. Swim plates (0.3% agar) were inoculated for two days at 25 °C. Images were taken 24- and 48-h post-inoculation. Values are the means ± standard deviations from four replicates. Two-way analysis of variance (ANOVA) test was performed. ** Significant difference (*p*-value < 0.01)

The T6SS has been implicated in pathogenicity [15, 52, 53], motility [54–56] and interaction with other bacteria [21, 57]. Motility contributes to virulence in the genus *Xanthomonas* [58–60]. We therefore tested the T6SS mutants for swimming motility on petri dish with a low proportion of agar (0,3% agar) after 24 h and 48 h. Motility was significantly different after 48 for the CIO151Δ*clpV* mutant with respect to the (*p*-value = 0.0007). Notably, the motility of this strain was indistinguishable from that of the CIO151 strain, after transformation with the wild type *clpV* gene (Fig. 5c), which demonstrates that the observed differences was due to the truncation/deletion of this gene. For the other evaluated mutants, there were no differences in motility with respect to the wild type (*p*-value> 0.05; Fig. 5a, b and d). In general, these results suggest that ClpV activity impacts motility, whereas VgrG*,* IcmF and Hcp are dispensable for this function in *Xpm*.

## Discussion

The T6SS is a versatile macromolecular assembly that has not been comprehensively characterized in plant pathogens. We have provided insights into the gene cluster organization and phylogeny of 15 genes of the T6SS machinery in the genus *Xanthomonas*. We found that the T6SS may have suffered duplications and HGT events in *Xanthomonas*. Finally, we show the importance of the T6SS for aggressiveness in susceptible cassava plants and in vitro motility of *Xpm*.

We detected the presence of three distinct T6SS clusters in the genus *Xanthomonas* that differ in the order in which the orthologous genes are arranged (Fig. 1 and Fig. S1). The number of T6SS clusters has also been found to be variable in other taxa [12]. The organization of the clusters I and III is similar, containing 16 genes, contrary to cluster II, with only 12 genes. Because the T6SS cluster II always co-occurs with one of the other clusters, cluster II may act as a complementary system in *Xoo*. This has previously been reported for other plant pathogens such as *B. glumae, B gladioli* and *B. plantari* strains, which generally possess at least two potential functional T6SS clusters whilst most representatives of beneficial (plant growth-promoting, symbionts or nodule-forming) *Burkholderia* species showed one or two clusters [61].

The presence of two T6SS in *X. oryzae*, *Xeu* and *Xp* could indicate that both clusters together are required to function properly. This is also the case of *P. aeruginosa,* where the cluster I, which is involved in virulence [15] and toxicity [1] requires the activity of clusters II and III [8]. Similarly, *Vibrio cholerae* has two auxiliary clusters with important activity during competition between *V. cholerae* strains [62]. Hence, it would be interesting to define the environments where individual T6SS clusters are active and conditionally essential in Xanthomonads.

Several lines of evidence suggested horizontal gene transfer events in the T6SS regions of the analyzed *Xanthomonas*. This observation agrees with previous phylogenetic studies, in other taxa, reporting the presence of T6SS gene clusters in horizontally acquired pathogenicity islands [12, 63]. The presence of insertion sequences and genomic islands in the T6SS cluster III, together with the disagreement between the genomes-based and T6SS-based phylogenetic relationships of the genus *Xanthomonas* [28] support the independent acquisition of the cluster III, by HGT. No differences were found in GC content were detected between the T6SS clusters and their corresponding genomes. This pattern of homogeneity in GC content was previously found in the T6SS of *Klebsiella* spp. and *V. cholerae* [64, 65]. Thus, we hypothesize that if the horizontal acquisition of the T6SS indeed happened in *Xanthomonas*, the horizontally acquired T6SS regions have adapted to the characteristics (GC content, codon usage, etc.) of the receiving genome to optimize its expression.

T6SS gene expression is tightly regulated at the transcriptional [by transcription factors (TFs)] and post-transcriptional levels (e.g. phosporylation). Cluster I showed a phosphorylation-type regulator (Kinase / Phosphatase / Forkhead). Cluster III has both a phosphorylation-type regulator and an AraC-type TF. Cluster II does not have either cis-acting regulator. Miyata et al. recently proposed that the presence of multiple regulators may help a pathogen coordinate T6SS gene expression to avoid identification by the host immune system [63]. A similar phenomenon could be occurring in Xanthomonads. Understanding the regulatory mechanisms of the T6SS in *Xanthomonas* will be a key step to uncover its role in diverse ecological interactions.

A single T6SS (with 16 genes and classified as type III*)* was identified in the *Xpm* CIO151 genome. We showed that the T6SS is an important factor for virulence and motility in this pathovar. *clpV* is divided in two contigous sequences in both *Xp* and *Xpm*. The importance of ClpV is species-specific. For example, ClpV supplies the energy for the assembly of the T6SS external machinery, composed of VipA (ImpB) and VipB (ImpD) in *V. cholerae* and *P. aeruginosa* species [14, 66]. However, the lack of ClpV does not affect the functionality of the T6SS of *Campylobacter jejuni* [67]. In *Xpm* CIO151, each *clpV* fragment contain a potentially functional ATPase domain and P-loop containing nucleoside triphosphate hydrolase, corresponding to a common ClpV protein. Using a Δ*clpV* mutant, we demonstrated that *clpV* is important for in vitro motility of *Xpm.* This finding suggests that the fragmented *clpV* gene is still functional. Motility plays a role predominantly in the early phases of infection; thus it helps in the development of plant disease in *Xanthomonas* [68]. *Xpm* probably requires motility for the pre-entry processes and the spread of the pathogen inside the plant, but no experimental tests have been performed to test this. To fully understand the role of *clpV* in motility, it would be important to fully characterize the regulation of motility in *Xpm* and what molecular role ClpV plays in it.

We demonstrate a decrease in virulence for the CIO151Δ*vgrG,* CIO151Δ*clpV* and CIO151Δ*hcp* mutants*.* Hcp has been reported as a substrate of the T6SS in *P. aeruginosa* [15] and *Burkholderia mallei* [21]*.* More importantly, Hcp was required for full tumorigenesis efficiency in *A. tumefaciens* [16], in agreement with our results for *Xpm*. Mougous and collaborators (2006) [15] demonstrated that ClpV is necessary for the secretion of Hcp in *P. aeruginosa*. In *Xpm*, we have demonstrated that ClpV has pleiotropic effects on two processes – motility and virulence. It is therefore possible that Hcp is a substrate of ClpV, affecting other aspects of virulence different from motility, and that other ClpV-dependent, Hcp- and VgrG-independent activities are required for motility in *Xpm*. In addition to Hcp- and VgrG, a few T6SS effectors have been reported until now, namely EvpP from *Edwardsiella tarda* [69], RbsB in *Rhizobium leguminosarum* [70, 71], TssM in *B. mallei* [21], and Tse1, Tse2, and Tse3 in *P. aeruginosa* [1, 72]. Recently, Bayer-Santos and collaborators (2019) in silico predicted a series of T6SS effectors from *Xanthomonadales* using an in silico analysis and suggested the presence of a high number of putative antibacterial toxins [73] which need to be experimentally tested. Bioinformatic analyses (using the Bastion6 software [74]) could be used to identify effector candidates for the T6SS in *Xpm.* Those predictions could be then experimentally evaluated in follow up experiments to better elucidate the distinct roles of each effector in the phenotypes observed here.

## Conclusions

Our computational analyses identify 16 proteins of the T6SS in the genus *Xanthomonas*. T6SS presents three different T6SS-associated gene clusters are present in the genus *Xanthomonas* that vary principally in the organization and the synteny of orthologous genes between species. Clusters III and I have the same number of genes and organization, while cluster II only has 12 genes, and it is restricted to *X. oryzae* strains. Phylogenetic analyses suggest that the T6SS might have been acquired by a very ancient event of horizontal gene transfer and maintained through evolution, hinting at their importance for the adaptation of *Xanthomonas* to their hosts. Finally, we demonstrated that the T6SS of *Xpm* is functional, and significantly contributes to virulence and motility.

## Supplementary Information

**Additional file 1: Table S1.** TBLASTN and BLASTP of homologous structural genes of *Pseudomonas aeruginosa* T6SS selected. Homologous structural genes of *Pseudomonas aeruginosa* T6SS selected by TBLASTN (Altschul et al., 1997) against the genomes of *Xanthomonas citri* subsp. *citri* str. 306 (*Xcc*), *Xanthomonas euvesicatoria* str. 85–10 (Xeu) and *Xanthomonas phaseoli* pv. *manihotis* str. CIO151 (*Xpm*). Similarity of structural genes of *Pseudomonas aeruginosa* T6SS selected by BLASTP against the genomes of *Xanthomonas oryzae* pv. oryzae str. PXO 099 (Xoo), *Xanthomonas campestris* pv. *campestris* str. ATCC 33913 (Xcac) and *Xanthomonas albilineans* GPE PC73 (Xalb). Colors follow the same guidelines as in Fig. 1. **Table S2**. Bacterial strains and plasmids used in this study. Derivative strains of *Xanthomonas phaseoli* pv. *manihotis* str. CIO151 (*Xpm*) with knockouts in T6SS genes. **Table S3.** List of primers used in this study. **Table S4.** Analysis of *Xanthomonas* genomes with or without T6SS genes. Results of BLASTP and ORTHOMCL analyses show that the reconstructed T6SS clusters in *Xanthomonas* contain between twelve and sixteen genes. **Table S5.** Structural genes of type VI secretion system of *Xanthomonas*. Distribution of type VI secretion system gene clusters in 14 strains of *Xanthomonas*. For each strain of *Xanthomonas*, the genes found in T6SS are divided by clusters (I, II and II) and are shown with gene, protein and product sizes. **Table S6.** Results of the search for T6SS-encoding genes in the genomes of *Xanthomonas perforans* (Xp 91–118), *Xanthomonas cassavae* str. CFBP4642 and *Xanthomonas axonopodis* str. 29 with EDGAR 2.0. EDGAR displays the orthologous genes in each genome. For each gene, the nomenclature and annotation are denoted. **Table S7.** Localization, protein family and motif prediction of T6SS proteins in *Xpm*. The core proteins of T6SS were subjected to in silico analyses for subcellular localization using PSORT, prediction of transmembrane domains (TMpred), domain prediction (INTERPRO, CDD and PRODOM) and protein families (Pfam) and motifs (Motif finder and MOTIF search). The colors follow the same guidelines as in Fig. 1. **Table S8**. Insertion sequences in the T6SS. The clusters of the T6SS of *Xeu*, *Xcc3* and *Xpm* were examined for IS families and groups, defined in the database ISfinder [31].

**Additional file 2: Fig. S1.** Genomic organization of the characterized T6SS cluster. Alignment orthologous sequences and the hits of T6SS genes are represented as colored arrows. The colors follow the same guidelines as in Fig. 1. A unique color was assigned to highly conserved orthologs of *Xanthomonas euvesicatoria* str. 85–10, *Xanthomonas citri* subsp. *citri* str. 306 and *Xanthomonas phaseoli* pv. *manihotis* str. CIO 151. The lines represent the orthologous genes found with ORTHOMCL.

## Data Availability

The datasets used and/or analyzed during the current study are available from the corresponding author on reasonable request.

## Abbreviations

T6SS: Type VI secretion system
*Xpm*: *Xanthomonas phaseoli* pv. *manihotis*
IcmF: Intracellular multiplication protein F
Hcp: Haemolysis-corregulated protein
VgrG: Valine-Glycine repeats G
COG: Cluster of orthologous groups
HGT: Horizontal Gene Transfer
IS: Insertion Sequence
*Xcc3*: *Xanthomonas citri* subsp. *citri* strain 306
*Xeu*: *Xanthomonas euvesicatoria* 85–10
*Xv*: *Xanthomonas vesicatoria* strain 1111
*Xp*: *Xanthomonas perforans* strain 91–118
*Xoo*: *Xanthomonas oryzae* pv. *oryzae*
*Xcac*: *Xanthomonas campestris* pv. *campestris*
*Xalb*: *Xanthomonas albilineans*
