# Supplementary material for: The type VI secretion system of Xanthomonas phaseoli pv. manihotis is involved in virulence and in vitro motility
Source: BMC Microbiol. 2021 Jan 6;21:14. doi: 10.1186/s12866-020-02066-1 (PMC7788950; doi:10.1186/s12866-020-02066-1)
Supplement: Supplementary file 2 — Additional file 2: Fig. S1. Genomic organization of the characterized T6SS cluster. Alignment orthologous sequences and the hits of T6SS genes are represented as colored arrows. The colors follow the same guidelines as in Fig. 1. A unique color was assigned to highly conserved orthologs of Xanthomonas euvesicatoria str. 85–10, Xanthomonas citri subsp. citri str. 306 and Xanthomonas phaseoli pv. manihotis str. CIO 151. The lines represent the orthologous genes found with ORTHOMCL. [file 12866_2020_2066_MOESM2_ESM.docx]

**Supplementary Figures**

**Figure S1. Genomic organization of the characterized T6SS cluster.** Alignment orthologous sequences and the hits of T6SS genes are represented as colored arrows. The colors follow the same guidelines as in Fig. 1. A unique color was assigned to highly conserved orthologs of *Xanthomonas euvesicatoria* str. 85-10, *Xanthomonas citri* subsp. *citri* str. 306 and *Xanthomonas phaseoli* pv. *manihotis* str. CIO 151. The lines represent the orthologous genes found with ORTHOMCL.
